# Unveiling the Uncommon: A Rare Case of Group A Streptococcus Axillary Lymphadenitis With Ambiguous Presentation Leading to Multiple Misdiagnoses

**DOI:** 10.7759/cureus.60806

**Published:** 2024-05-21

**Authors:** Geovanna Badaro, Matthew Passeggiata, Nicole Casio, David Thomas, Elias Shattahi

**Affiliations:** 1 General Surgery, Trumbull Regional Medical Center, Warren, USA; 2 General Surgery, Sharon Regional Medical Center, Sharon, USA; 3 Infectious Disease, Sharon Regional Medical Center, Sharon, USA

**Keywords:** abscess, puncture wound, suppurative lymphadenitis, axillary lymphadenitis, group a streptococcus pyogenes

## Abstract

Axillary lymphadenitis in adults presents a diagnostic challenge due to its diverse etiology and variable clinical manifestations. We present a rare case of suppurative Group A *Streptococcus* (GAS) axillary lymphadenitis secondary to a puncture wound, emphasizing the critical importance of differential diagnosis and immediate intervention. A 36-year-old male initially presented with left axillary pain and discomfort following a traumatic injury to the left thumb. Despite multiple healthcare encounters and misdiagnoses including viral illness and shingles, the patient's condition deteriorated, manifesting as fever, edema, and erythema in the left axilla. This case underscores the paramount significance of considering lymphadenitis in patients with axillary symptoms, particularly following trauma or skin breaches. Early recognition and appropriate management are crucial to prevent grave complications such as abscess formation, thrombophlebitis, and bacteremia. Streptococcal axillary lymphadenitis should be included at the forefront of the differential diagnosis to expedite treatment and mitigate potential life-threatening consequences associated with delayed diagnosis.

## Introduction

The lymph nodes function as an important arm of the immune system, filtering infectious agents and responding to foreign bodies by activating lymphocytes and white blood cells. Lymph nodes in general can be categorized as superficial or deep lymph nodes, depending on location within subcutaneous tissues (superficial) or within the deep fascia (deep). In relation to the upper limb, the superficial lymph nodes all drain into the deep axillary lymph nodes. Infections of any part of the hand or arm drain upward towards the axilla [1]. The axillary lymph nodes receive blood supply from the axillary artery and drainage from the axillary veins [2]. While lymph nodes work to recruit and proliferate immune cells due to infectious, inflammatory, or malignant processes, they are susceptible to inflammation [3].

Lymphadenitis is the inflammation of lymph nodes, which can be characterized acutely or chronically in a single lymph node or in a larger anatomical region. The most common culprits of lymphadenitis include skin infections, viruses, and atypical bacterial disease. Common infectious causes of lymphadenitis include *Staphylococcus* and *Streptococcus* species, cat-scratch disease, tuberculosis, HIV, Epstein-Barr virus (EBV), and cytomegalovirus (CMV); expectedly due to lymphatic spread [4,5].

Immunocompromised individuals are most at risk for lymphadenitis, as normal flora is most likely to dominate a suppressed immune system and subsequently irritate lymph pockets. In the case of HIV/AIDS, immune cells get destroyed, leaving very little to mount an adequate immune response to foreign pathogens. In autoimmune disease, immune cells are unable to recognize which is self and which is foreign, causing damage to healthy cells and tissues, and impacting the ability of an immune response. In certain medical treatments, such as cancer treatments or the use of corticosteroids, immune cells may be suppressed or damaged, leading to a depletion of antibodies to create a proper immune response to pathogens [6].

Rare occasions of lymphadenitis in immunocompetent individuals have widely gone without being studied despite the painful prodrome and high risk of bacteremia due to lymphatic or hematologic spread [4,5,7]. Historically, lymphadenitis has been mainly reported as a part of disease sequelae, in specific cases of tuberculous and non-tuberculous mycobacterium, consequence of animal microbiota in animal handlers, and as a rare differential of cervical lymphadenopathy in children [8,9]. However, few cases of localized lymphadenitis have been reported and investigated. This report describes the rare and severe case of streptococcal axillary lymphadenitis due to Group A *Streptococcus* (GAS).

## Case presentation

**Figure 1:**
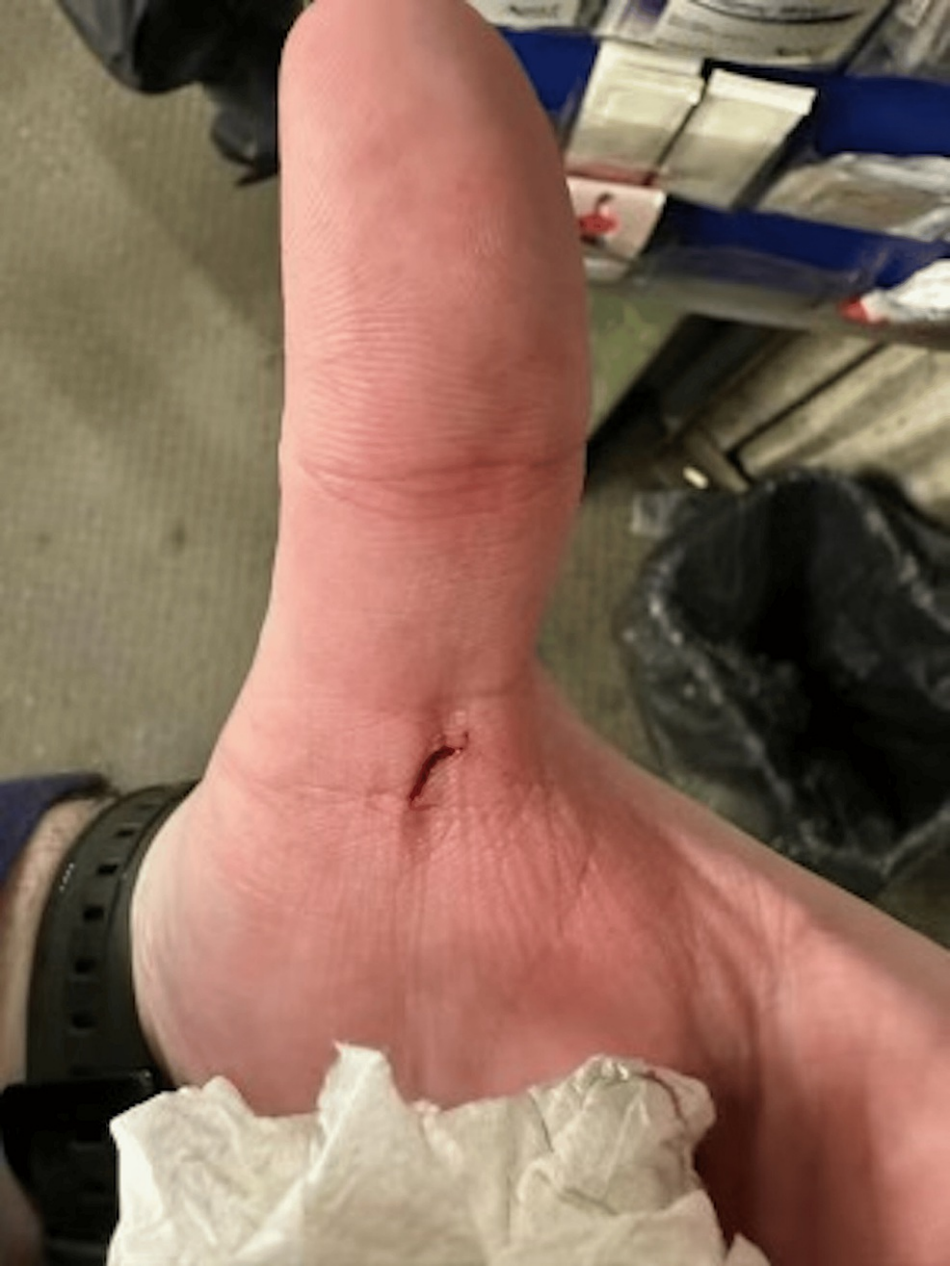
Puncture wound to the base of the dorsal aspect of the left thumb.

A 36-year-old male with a history of gout presented to the Sharon Regional Medical Center Emergency Department in Sharon, Pennsylvania, with left axillary pain and discomfort. Six weeks prior to these initial symptoms, he had an accident at work where a piece of galvanized steel with multiple chemicals on it punctured the base of the dorsal aspect of the left thumb (Figure 1). The patient described the wound to be as deep as a Q-tip head. He was evaluated at the work clinic, Steri-Strips (3M Company, Saint Paul, Minnesota, United States) were placed, and a tetanus shot was administered.

A week after the initial wound, the patient was evaluated in the emergency department (ED) after developing fever, chills, shortness of breath, productive cough, headache, and left shoulder pain. At this evaluation, the patient tested negative for both coronavirus disease 2019 (COVID-19) and Influenza virus. A chest X-ray was done revealing no significant findings. The patient was diagnosed with a viral illness and discharged home.

Three days after the previous ED encounter, he woke up with a red rash, which he described as the size of his hand, on the lateral side of his left chest wall. He went to an urgent care center to be evaluated and was diagnosed with shingles. He was treated with valacyclovir and gabapentin, which he took for seven days, and the rash seemed to have dissipated, according to the patient. 

**Figure 2:**
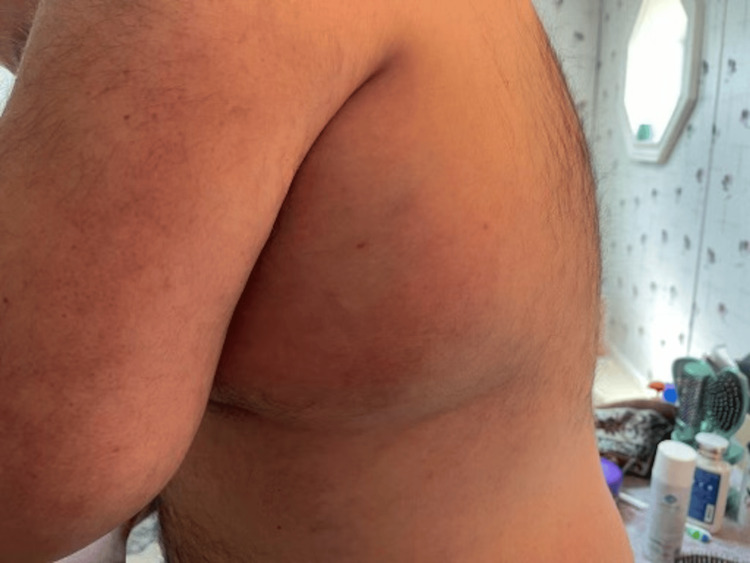
Moderate erythema and edema below the left axilla where a rash was previously located.

Twelve days after being diagnosed with shingles, he followed up with his primary care physician (PCP). During this visit, the patient endorsed a cough which was then evaluated with another chest X-ray, which was also unremarkable. Blood cultures were obtained as well as a Lyme disease serology test. The patient was referred to an orthopedic surgeon for continued left shoulder pain, discomfort, and limited range of motion. Two weeks after his visit with his PCP, he developed another episode of high fever, sweats, and edema below his left axilla where the rash was previously located (Figure 2). The patient’s symptoms progressively worsened over the next few days which brought him into the ED. During this visit, general surgery was consulted for evaluation of the left axilla swelling and discomfort. On examination, there was moderate edema of the upper left chest and left axilla, moderate erythema, fluctuance, as well as warmth to touch, and moderate-to-severe tenderness to palpation. 

**Figure 3:**
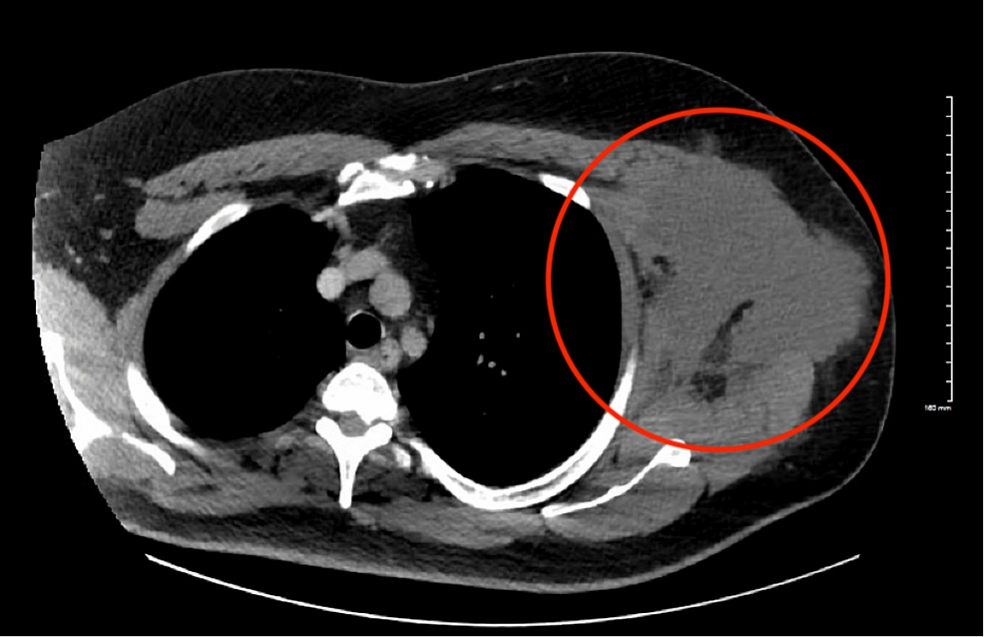
CT (axial view) of the chest showing a large soft tissue density mass (red circle) involving the left chest wall and axillary region measuring 14x10 cm

**Figure 4:**
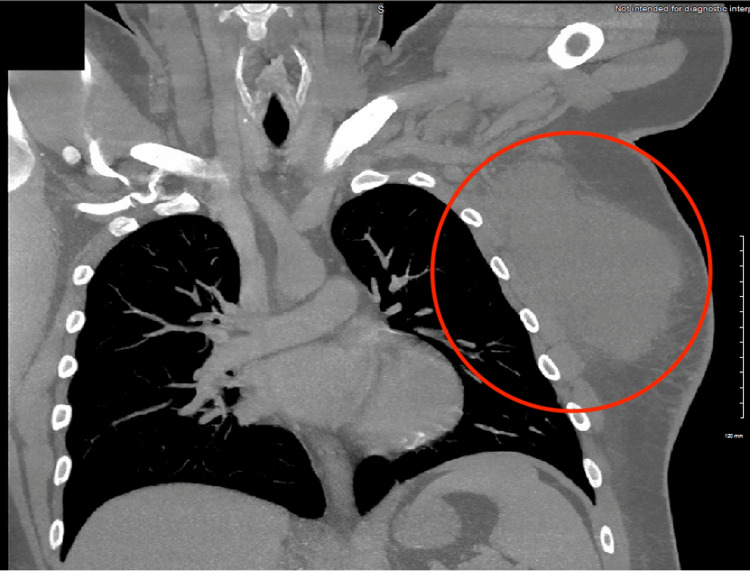
CT (coronal view) of the chest showing a large soft tissue density mass (red circle) involving the left chest wall and axillary region

Laboratory tests were obtained and initial reports showed an increased white blood cell count of 14.8 and an increased erythrocyte sedimentation rate (ESR) of 135. Computed tomography (CT) imaging of the chest wall revealed a large soft tissue fluid collection involving the left lateral chest wall and left axillary region, measuring 14 x 10 cm (Figures 3, 4). Due to the clinical presentation, the diagnosis of an abscess was made. The patient was scheduled for an incision and drainage of the abscess in the operating room where about 500 cc of malodorous, white-greenish purulent fluid, was obtained and sent for culture. A counter incision was made with the placement of a Penrose drain for continuous drainage.

The initial Gram stain results revealed gram-positive cocci in chains, suggestive of streptococcal infection. The patient was started on ampicillin-sulbactam plus vancomycin pending mature culture results. Vancomycin was to be discontinued if methicillin-resistant *Staphylococcus aureus* (MRSA) was negative. The final deep wound culture revealed predominant growth of GAS (*Streptococcus pyogenes*) and rare growth of MRSA. Postoperatively, the patient reported feeling better; he was afebrile and his white blood cell count had returned to normal limits.

In this case, we performed surgical drainage of the abscess, and the patient was discharged home on linezolid, 600 mg tablets every 12 hours for two weeks due to the slight growth of MRSA and moderate growth of GAS. The patient was also scheduled for a follow-up visit at the surgery clinic before the completion of antibiotic treatment. The Lyme disease IgG and IgM serology titers were both negative. The patient was doing well at his follow-up visit and there were no further complications.

## Discussion

The lymph nodes of the hand drain into the axillary lymph nodes. The thumb in particular drains to the pectoral lymph nodes in the medial axilla [3,4], resulting in this patient’s axillary lymphadenopathy secondary to penetrating injury in the left thumb. The index finger drains to the central axillary lymph nodes at the axilla base, the middle finger to the apical axillary, the ring finger to the lateral axillary, and the little finger to the subscapular posterior axillary [10]. In this case, this was how the thumb trauma led to axillary lymphadenitis.

The most common causative pathogens consist of normal skin flora, *S. aureus*, and *S. pyogenes* with traumatic access combined with patient immunodeficiency making them susceptible to infection. Other etiologies include *Bartonella henselae, Mycobacterium *species, EBV, and CMV [5,8-12]. However, at certain ages, patients are more at risk to certain pathogens causing lymphadenitis due to immature immunity. One study showed suppurative lymphadenitis in children to be caused by gram-positive bacteria in 66.7% of cases, most commonly consisting of *S. aureus, S. pyogenes, and M. tuberculosis* [13]. While acute lymphadenitis is a rare condition, it is more common in children than adults due to either an underlying immune condition or an immature immune response. Axillary lymphadenitis rarely presents alone and is more often accompanied by cervical lymphadenopathy [14].

Other important patient populations to consider axillary lymphadenitis in are immunocompromised individuals, particularly those with HIV infection or patients undergoing immunosuppressive regimens. They are at the highest risk for lymphadenitis due to their increased risk of infection which could easily drain and infect lymph nodes [15,16].

Axillary lymphadenopathy is a particularly challenging case for clinicians, as lymphadenopathy is commonly a symptom of a disease, rather than the actual diagnosis. With the presentation of axillary lymphadenopathy, malignancy of the breast, lung, or leukemia/lymphoma must always be considered a cannot-miss differential. After ruling out malignancy, many other differentials are left to be considered. The lymphadenopathy may be a reactive immune process to a benign breast pathology like mastitis, or a recent viral illness or vaccine. Autoimmune diseases like systemic lupus erythematosus, rheumatoid arthritis, HIV, and granulomatous diseases like sarcoidosis can also cause axillary lymphadenopathy. 

On first evaluation in the ED, one week after his work incident, the patient’s chief complaint was a cough. The focus on his acute fever and respiratory symptoms drove the differential diagnoses as pneumonia, lung malignancy, or viral illness, which was evaluated first with a chest x-ray. The diagnostic concern was for pneumonia and without pulmonary infiltrates or opacities, the diagnosis of viral respiratory illness was made. His shoulder pain was dismissed as secondary to coughing and the axillary lymphadenitis was missed. 

Ten days after the patient’s initial incident, his worsening symptoms and new onset rash led him to seek further care. Here the clinician focused on the patient’s chief complaint of new-onset skin rash. Consider the clinical picture: a patient with recent viral illness, acute nonspecific symptoms of fever, malaise, and new-onset skin rash limited to a specific area of the chest. The dermatomal rash of shingles certainly seems like the probable culprit, with other running differentials being contact dermatitis or an autoimmune blistering rash. Once more, the lymphadenitis was overlooked, this time as a symptom of shingles. 

At the patient’s visit with his PCP, the clinical picture of a patient in the Pennsylvania area with acute onset of fever, myalgia, cough, joint pain, and rash with little to no relief with previous therapies of antivirals and gabapentin, began to point towards other etiologies. One differential that comes to mind is Lyme disease, which the clinician followed through with a Lyme serology test. The clinician further investigated the respiratory symptoms with a follow-up chest x-ray, with the possibility in mind that something may have changed since the initial ED visit. However, the left shoulder pain was thought to be of an orthopedic etiology. 

It is always a possibility that patients may not vocalize a symptom in the way clinicians expect. If this patient in particular reported shoulder pain, rather than axillary pain or colloquially armpit pain, a clinician who is dealing with a multifaceted case may not palpate the axilla or obtain an ultrasound to view the axillary lymph nodes. Particularly in this case, where the patient was involved with manual labor, arthritis or adhesive capsulitis may have been thought to be a much more likely culprit. Additionally, patients may not realize a lymph node is enlarged if the enlargement is subtle, therefore not mentioning any changes.

Including axillary lymphadenitis in the list of differentials for axillary pain and lymphadenopathy is important to be able to recognize, treat, and avoid unnecessary procedures as well as mental distress to patients [2,3]. However, if infectious pathology is overlooked for concerns over malignancy, patients may undergo unnecessary biopsies and procedures all while still suffering from the clinical prodrome of lymphadenitis [7,9,12,17]. Axillary lymphadenitis, while rare, was not considered a differential diagnosis until the patient’s final visit to the ED. At this visit, the patient endorsed high fever, sweats, and edema underneath the left axilla where a rash was previously located. The patient’s moderate edema and fluctuance led to consultation with General Surgery leading to the collection of bloodwork revealing a masked infectious process via the patient’s elevated white blood cell count and increased ESR and imaging revealing a large soft tissue fluid collection which was deemed to be an abscess due to the clinical presentation.

To properly evaluate axillary lymphadenopathy and begin to tackle the many differentials, Maini and Nagalli suggests starting with laboratory evaluation, including complete blood chemistry, complete metabolic panel, lactate dehydrogenase, fungal serologies, evaluation for syphilis, HIV, CMV, EBV, herpes simplex virus (HSV), hepatitis B virus (HBV), and QuantiFERON (QIAGEN N.V., Hilden, Germany) for tuberculosis, obtaining imaging such as CT, and, lastly, excisional node biopsy which is the gold standard for diagnosis [18].

Treatment should always include empiric antibiotic coverage of both gram-positive and gram-negative bacterium, and potentially adding coverage of MRSA [19]. In the case of this patient, the combination of culture-positive GAS and possible MRSA led to treatment with ampicillin/sulbactam and vancomycin until the cultures were matured. However, treatment can be tweaked depending on the patient's symptom severity and monitored closely for resolution. For example, short-term steroids may be needed if the patient is severely uncomfortable or in pain due to adenopathy. Additionally, should pain be severe or antibiotic treatment be inadequate, surgical drainage with continued broadened antibiotic coverage should be performed [20].

Complications of untreated lymphadenitis include consequences of continued inflammation spreading to other structures such as nearby veins. Inflammation to veins causes thrombophlebitis and blood clots to form, which can put a patient at increased risk of deep vein thrombosis, pulmonary embolism, septic thrombophlebitis, or stroke. Additionally, should a patient experience thrombophlebitis, the damage to the veins can lead to chronic venous insufficiency. Furthermore, there are the consequences of continued infection from undiagnosed lymphadenitis such as bacteremia and sepsis which can easily spread through the lymphatic system to other parts of the body, affecting multi-organ systems [9,12].

## Conclusions

We discussed a rare case of suppurative GAS axillary lymphadenitis secondary to a puncture wound. Prior to the patient’s final diagnosis, he was misdiagnosed with a viral illness, shingles, and musculoskeletal pain secondary to shoulder injury. It was not until 35 days after the patient’s initial visit to the ER, followed by three outpatient visits that he was diagnosed with suppurative axillary lymphadenitis. It is important for clinicians to have streptococcal axillary lymphadenitis as a differential diagnosis in any patient presenting with lateral chest wall rash, pain, fever after a trauma, impetigo, varicella, or any other skin breeches to obtain cultures in order to properly treat patients and prevent abscess formation and necrotizing lymphadenopathy. This early diagnosis and treatment can prevent serious complications of axillary lymphadenitis such as thrombophlebitis of the axillary or subclavian veins, pleural effusion, and bacteremia.
